# A comprehensive metabolomic dataset of human synovial fluid in osteoarthritis and healthy controls

**DOI:** 10.1016/j.dib.2025.112224

**Published:** 2025-11-07

**Authors:** Ping Chen, Shuai Zhang, Long Wu, Zhiyao Yong, Qunhua Jin, Kening Sun

**Affiliations:** a General Hospital of Ningxia Medical University, Yinchuan 750004, China; b Ningxia Key Laboratory of Clinical and Pathogenic Microbiology, General Hospital of Ningxia Medical University, Yinchuan 750004, China; c Orthopedics Ward 3, The General Hospital of Ningxia Medical University, Yinchuan 750004, China

**Keywords:** Osteoarthritis, Synovial fluid, Untargeted metabolomics, Biomarkers, UHPLC-MS

## Abstract

Osteoarthritis (OA) is a prevalent degenerative joint disorder that causes chronic pain and disability. The synovial fluid (SF), which bathes the joint, reflects the pathological state and is a valuable source for biomarker discovery. However, a comprehensive metabolomic profile of SF in OA remains to be fully established. Here, we present a comprehensive metabolomic dataset derived from SF samples of 10 OA patients and 10 matched healthy controls. Using non-targeted metabolomics based on ultra-high-performance liquid chromatography coupled with high-resolution mass spectrometry (UHPLC-MS), we identified 23,232 features, with 1431 metabolites annotated. Comparative analysis revealed 163 significantly differential metabolites (118 upregulated, 45 downregulated). Functional enrichment and pathway analyses highlighted disruptions in amino acid metabolism, energy metabolism, and lipid pathways. Several metabolites showed high diagnostic potential in ROC analysis. This dataset provides a valuable resource for understanding OA-associated metabolic alterations and offers robust candidate biomarkers for OA diagnosis and therapeutic monitoring.

Specifications Table

**Table:** 

Subject	Health Sciences, Medical Sciences & Pharmacology
Specific subject area	Osteoarthritis pathology,Biomarker Discovery, Dysregulated Metabolism
Type of data	Table, Graph, Figure Raw, Analyzed, Filtered
Data collection	Human synovial fluid samples were analyzed using LC-MS-based untargeted metabolomics, with metabolite identification performed against the BiotreeDB database. The study employed rigorous quality control protocols including pooled QC samples and technical replicates to ensure data reliability
Data source location	Ningxia Medical University General Hospital, Ningxia, China
Data accessibility	Repository name: Mendeley Data Data identification number: DOI: 10.17632/yhjnd9svtv.1 Direct URL to data: https://data.mendeley.com/datasets/yhjnd9svtv/1
Related research article	None

## 1 Value of the Data

• This dataset provides a detailed metabolic reference for human synovial fluid in osteoarthritis (OA), identifying 163 significantly altered metabolites compared to healthy controls.
• The data offers a foundation for biomarker discovery, as several differential metabolites show high diagnostic potential for OA in ROC analysis.
• Researchers can use this resource to explore perturbed metabolic pathways (e.g., amino acid and lipid metabolism) in OA pathogenesis and to compare with other joint diseases.

## 2 Background

Osteoarthritis (OA) affects over 300 million individuals worldwide and is a leading cause of disability in aging populations [1,2]. Pathologically, OA is characterized by cartilage degeneration, subchondral bone sclerosis, osteophyte formation, and synovial inflammation [3,4]. Despite its high prevalence, the underlying molecular and metabolic mechanisms remain incompletely understood. The synovial fluid (SF), as a key component of the joint cavity, bathes the cartilage and synovium, and its composition is believed to directly reflect the pathophysiological state of the joint. Therefore, SF represents an ideal biofluid for investigating OA-specific metabolic alterations. Metabolomics, as a post-genomic systems biology tool, provides a powerful approach to characterize small-molecule perturbations associated with disease progression [[5], [6], [7]]. However, comprehensive metabolomic profiling of SF in OA, particularly in comparison to healthy controls, remains limited. Here, we applied non-targeted metabolomics to profile synovial fluid alterations in OA patients compared with healthy controls, aiming to establish a comprehensive metabolic dataset and identify potential biomarkers and dysregulated pathways involved in OA.

## 3 Data Description

**Fig. 1:**
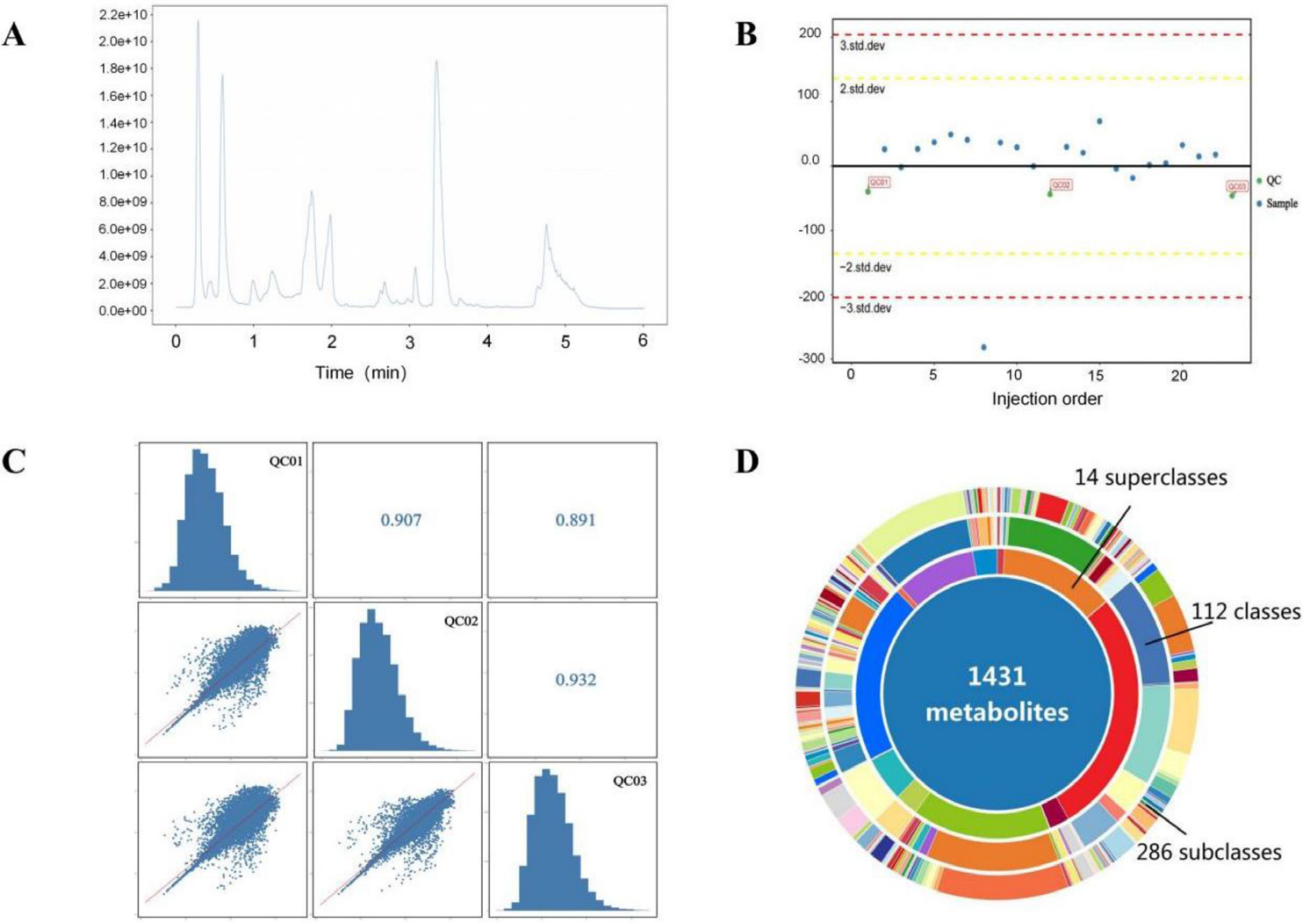
QC and multivariate statistical analysis of metabolomic data. (A) TIC diagram of positive ion mode in QC sample. (B) PCA-X one-dimensional distribution diagram of QC samples. (C) Correlation analysis of QC samples. (D) Classification ring diagram of metabolites.

**Fig. 2:**
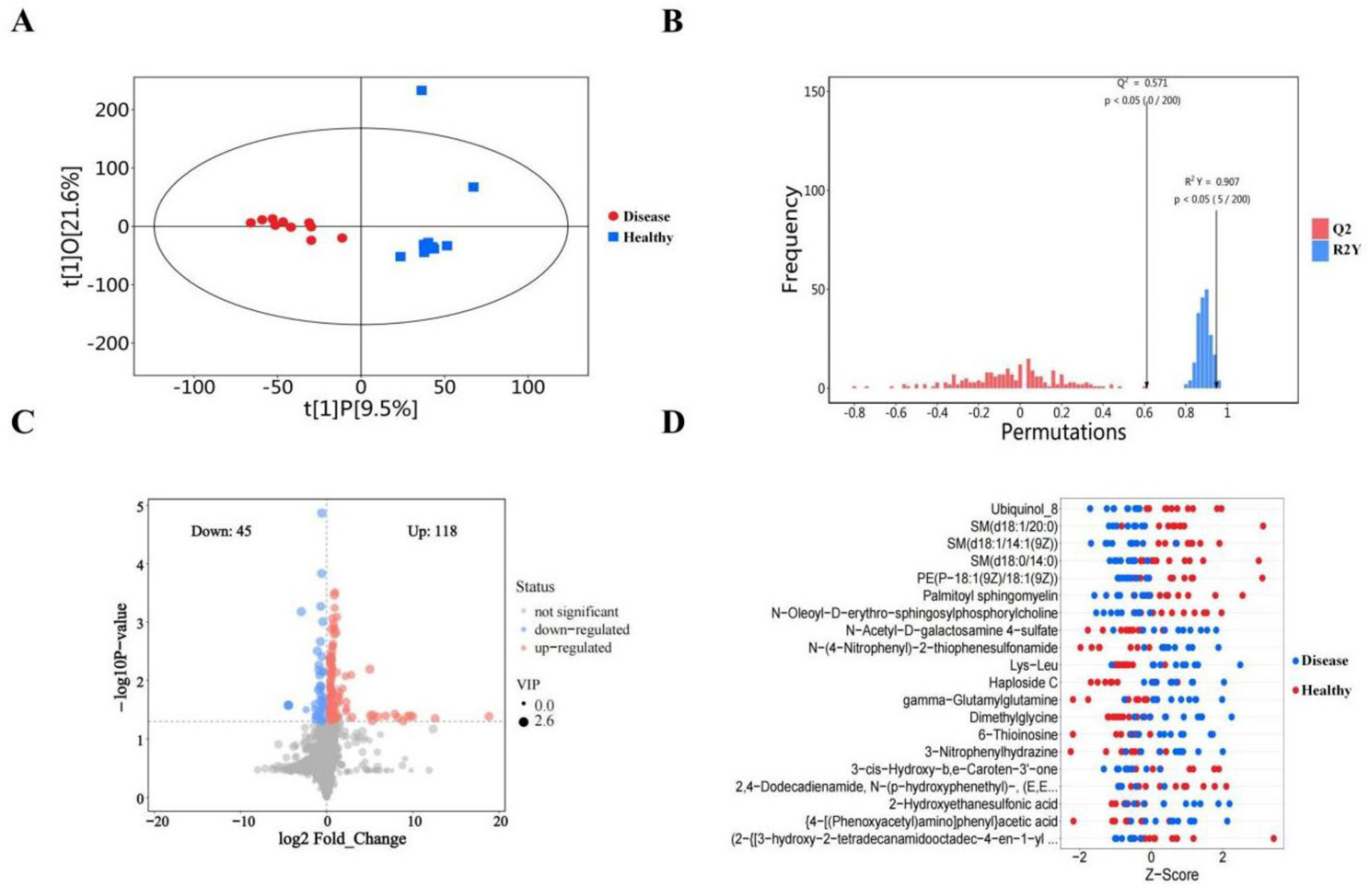
Identification of differential metabolites between osteoarthritis and healthy controls. (A) Scatter chart of OPLS-DA model score of healthy people versus diseased people. (B) Histogram of the permutation test results of healthy people versus diseased people. (C) Volcano plot of differential metabolite screening between healthy and diseased people. (D) Z-score diagram of differential metabolite screening between healthy and diseased people.

**Fig. 3:**
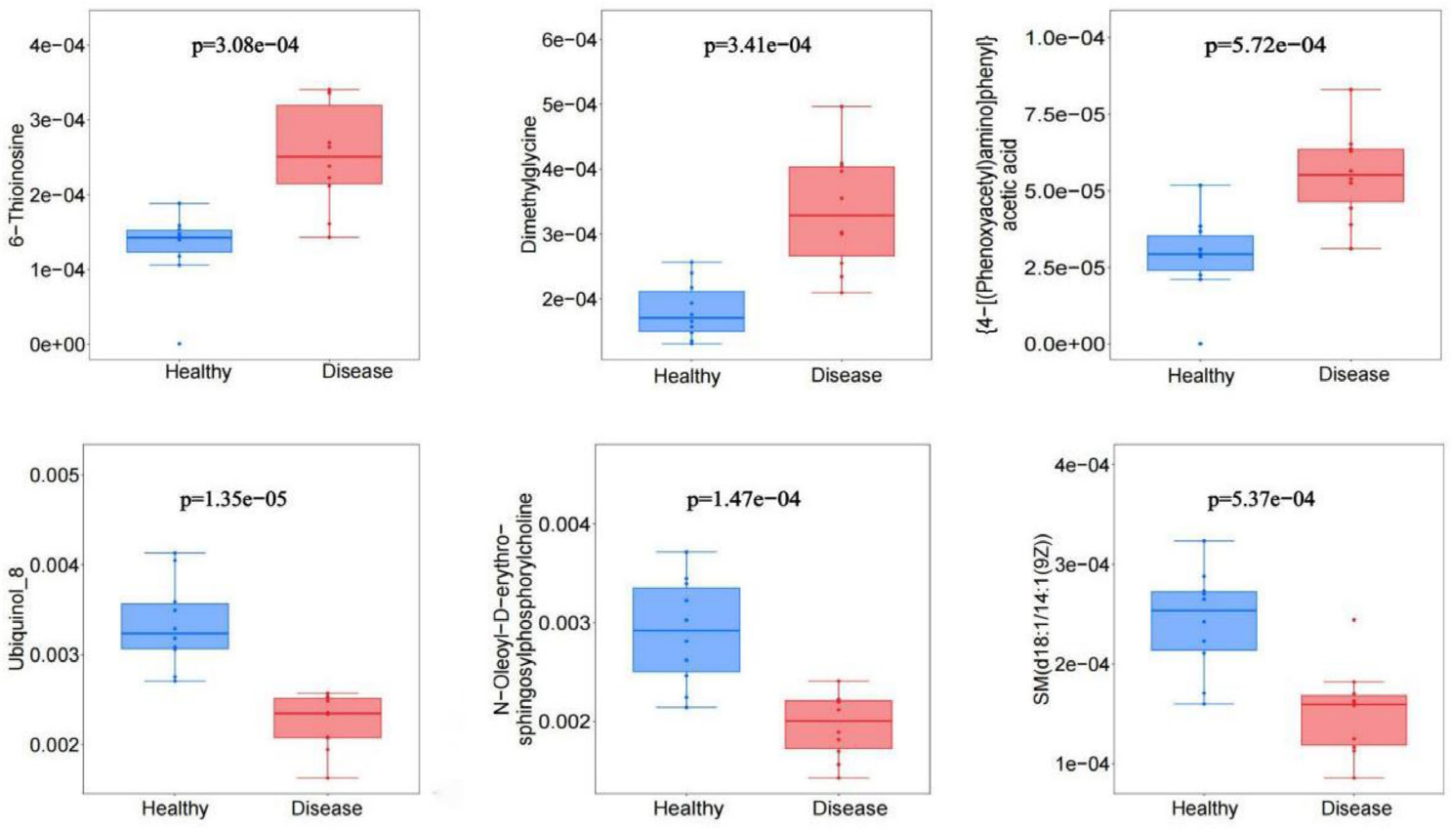
Boxplot analysis summarizing fold changes of key metabolites.

**Fig. 4:**
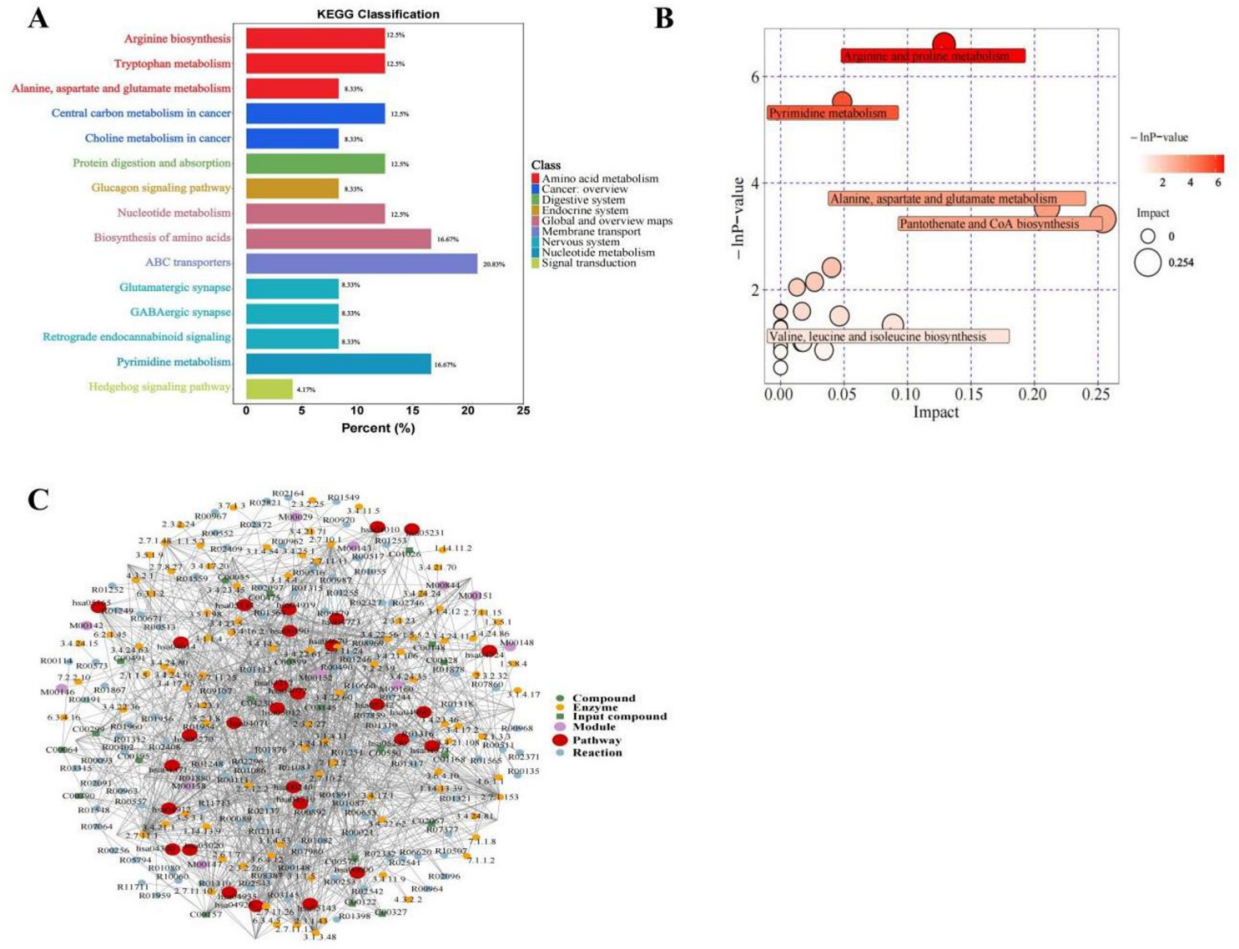
Metabolic pathway analysis of differential metabolites. (A) KEGG classification diagram of differential metabolites. (B) Bubble plot of differential metabolites. (C) Regulatory network analysis of differential metabolites.

**Fig. 5:**
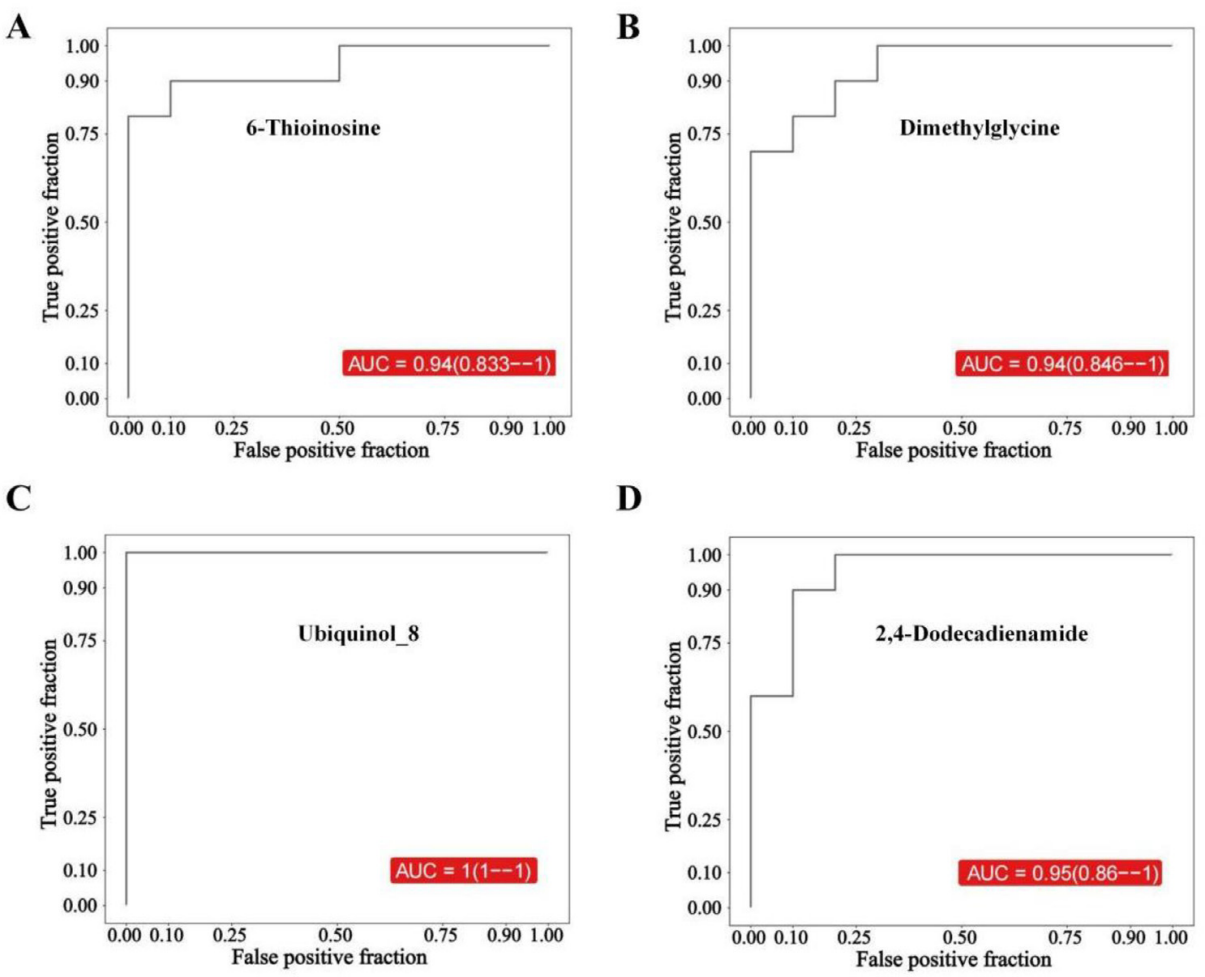
ROC curve analysis of representative upregulated and downregulated metabolites.

The quality and robustness of the acquired metabolomic data were rigorously assessed prior to biological interpretation (Table 1). Total ion chromatogram (TIC) analysis of quality control (QC) samples demonstrated high instrumental stability throughout the acquisition sequence (Fig. 1A). Unsupervised principal component analysis (PCA) revealed tight clustering of QC samples (Fig. 1B), while high inter-QC correlation coefficients further confirmed excellent data reproducibility (Fig. 1C). Metabolite classification analysis illustrated the broad chemical diversity of detected compounds (Fig. 1D). To maximize separation between osteoarthritis (OA) and healthy control (HC) groups, supervised orthogonal partial least squares-discriminant analysis (OPLS-DA) was employed. The score plot showed clear metabolic distinction between groups (Fig. 2A, Table 2), with permutation testing confirming model validity without overfitting (Fig. 2B). Differential metabolite screening identified 163 significantly altered metabolites, as visualized through volcano plot analysis based on statistical significance and fold-change thresholds (Fig. 2C, Table 3). A Z-score heatmap illustrated the abundance patterns of these differential metabolites across individual samples (Fig. 2D). The magnitude and direction of change for key metabolites were further detailed through boxplot analysis (Fig. 3). To elucidate the biological implications of these metabolic alterations, pathway enrichment analysis was performed. KEGG classification revealed the predominant categories of disturbed metabolites (Fig. 4A), bubble chart analysis highlighted the most significantly enriched metabolic pathways (Fig. 4B), and network visualization depicted interrelationships among differential metabolites (Fig. 4C). Finally, receiver operating characteristic (ROC) curve analysis was conducted to evaluate the diagnostic potential of representative upregulated and downregulated metabolites, several of which exhibited high sensitivity and specificity for distinguishing OA patients from healthy controls (Fig. 5).Collectively, these data establish a robust metabolomic dataset, validate data quality, characterize differential metabolic profiles between OA and controls, and highlight candidate biomarkers and pathways of biological relevance.

## 4 Experimental Design, Materials and Methods

### 4.1 Sample collection and preparation

Synovial fluid (SF) samples were obtained from 10 patients clinically diagnosed with osteoarthritis (OA) at Ningxia Medical University General Hospital and from 10 age- and sex-matched healthy controls undergoing arthroscopic procedures for non-OA related injuries (e.g., meniscal tear without cartilage damage). Inclusion criteria for OA patients followed the American College of Rheumatology guidelines, while exclusion criteria included rheumatoid arthritis, gout, infection, or metabolic disorders. Approximately 1–2 mL of SF was aspirated from the knee joint under sterile conditions and immediately centrifuged at 3000 × *g* for 10 min at 4 °C to remove cells and debris. The clarified supernatant was aliquoted into cryotubes and stored at −80 °C until further analysis.

### 4.2 Metabolite extraction

100 µL of thawed synovial fluid was mixed with 400 µL of ice-cold methanol/acetonitrile (1:1, v/v) containing a mixture of isotopically labeled internal standards (e.g., l-leucine-13C6, Cambridge Isotope Laboratories, Inc.). The mixture was vortexed for 1 min, incubated on ice for 10 min, and centrifuged at 12,000 × *g* for 15 min at 4 °C. The resulting supernatant was transferred into a fresh tube and dried under vacuum. Samples were reconstituted in 100 µL of 50 % acetonitrile in water, vortexed, centrifuged again to remove particulates, and transferred to LC vials for instrumental analysis. Quality control (QC) samples were prepared by pooling equal aliquots of all extracts and injected periodically to monitor instrument stability.

### 4.3 UHPLC—HRMS analysis

Metabolite separation was achieved using a Waters ACQUITY UPLC BEH Amide column (2.1 × 50 mm, 1.7 µm) on a Vanquish UHPLC system, with a gradient elution of 25 mM ammonium acetate/ammonia (pH 9.75, mobile phase A) and acetonitrile (mobile phase B) over a 20-min run time. The Orbitrap Exploris 120 mass spectrometer operated in dual-polarity mode (positive/negative ESI) with high-resolution settings (60,000 MS1, 15,000 MS2 resolution) and a mass range of *m/z* 300–1500. Key parameters included a sheath gas flow of 50 arb, auxiliary gas at 15 arb, capillary temperature of 320 °C, and spray voltages of ±3.4 kV. Data-dependent acquisition (DIA) was employed with staggered collision energies (20/30/40 eV) to maximize metabolite coverage and fragmentation quality. System performance was validated through daily calibration with Pierce FlexMix standards and continuous monitoring of retention time drift (<0.1 min) and mass accuracy (<2 ppm) using QC samples.

### 4.4 Data processing

Raw LC-MS files were converted into mzXML format using ProteoWizard software (version 3.0) [8]. Peak picking, retention time alignment, and signal integration were conducted with BiotreeDB (version 3.0). Metabolite annotation was performed by matching accurate mass, retention time, and MS/MS spectra against internal standards and the BiotreeDB spectral library. Metabolite identification confidence was classified according to the Metabolomics Standards Initiative (MSI) guidelines, with putative annotations (Level 2) assigned based on MS/MS spectral matching. QC-based robust LOESS signal correction (QC-RLSC) was applied using the statTarget package in R to minimize signal drift across the batch. Features detected in <50 % of QC samples or with a relative standard deviation (RSD) >30 % were excluded from further analysis.

### 4.5 Statistical analysis

Normalized intensity data were subjected to multivariate and univariate statistical analyses using R (v4.2.0) and SIMCA-P (v16.0). Principal component analysis (PCA) was used to visualize overall sample distribution and identify clustering trends. Orthogonal partial least squares discriminant analysis (OPLS-DA) was performed to maximize group separation, with model quality assessed by R2Y and Q2 values. A permutation test (200 permutations) was conducted to guard against overfitting. Statistical significance was evaluated using Student's *t*-test (*p* < 0.05) combined with fold-change (|log2FC|≥1) thresholds. The false discovery rate (FDR) was controlled using the Benjamini-Hochberg method. Variable importance in projection (VIP) scores >1 from OPLS-DA models were also applied to prioritize significant metabolites. Volcano plots, heatmaps, and boxplots were generated to illustrate metabolite variations.

### 4.6 Bioinformatics

Differential metabolites were subjected to functional clustering and pathway enrichment analysis using MetaboAnalyst 5.0 [9,10]. Annotation databases included KEGG and HMDB. Enrichment analyses identified perturbed pathways with a pathway impact value >0.1 and p 〈 0.05 considered significant. Correlation network analysis was performed to visualize interactions among metabolites using a correlation threshold of |r| 〉 0.7 and *p* < 0.05.

## Limitations

While this study provides comprehensive metabolomic profiling of OA synovial fluid, several limitations should be noted. First, the sample size was relatively small (10 OA and 10 controls), which may limit statistical power and generalizability. Second, only synovial fluid metabolites were profiled, without integration of other omics layers (e.g., transcriptomics or proteomics), thereby restricting mechanistic interpretation. Third, although LC-MS provides high sensitivity, metabolite annotation was based on spectral libraries and putative identifications; further validation with authentic standards is needed. Finally, the single-center recruitment from Northwest China may affect generalizability to other ethnic populations. Future studies should incorporate longitudinal sampling and multi-omics integration to address these constraints.

## Ethics Statement

The study was conducted in accordance with the Declaration of Helsinki and approved by the Ethics Committee of the General Hospital of Ningxia Medical University (Ethical Approval No 2020–166).

## Credit Author Statement

**Ping Chen:** Conceptualization, Data curation, Methodology, Writing-original draft; **Shuai Zhang:** Data curation, Methodology, Writing-original draft; **Long Wu:** Methodology, Software; **Zhiyao Yong:** Investigation, Validation; **Qunhua Jin:** Conceptualization, Supervision, Writing-review and editing; **Kening Sun:** Conceptualization, Funding acquisition, Investigation, Project administration, Supervision, Writing-original draft, Writing-review and editing.

## Data Availability

Mendeley DataA comprehensive metabolomic dataset of human synovial fluid in osteoarthritis and healthy controls (Original data).

## Appendix Supplementary materials

Image, application 1

Image, application 2

Image, application 3
